# Under Pressure: Negative Urgency and Disordered Eating in the Context of Parental Differential Support

**DOI:** 10.1111/jmft.70159

**Published:** 2026-07-28

**Authors:** Hamide Gozu, Jenna K. Rieder

**Affiliations:** ^1^ College of Humanities & Science, Psychology Thomas Jefferson University Philadelphia Pennsylvania USA

**Keywords:** binge eating, compensatory behaviors, negative urgency, parental differential support, weight‐shape concerns

## Abstract

Disordered eating behaviors are prevalent in emerging adulthood and may be shaped by family dynamics. This study examined associations among maternal and paternal differential support, weight–shape concerns, and binge eating and compensatory behaviors in emerging adulthood, while testing the moderating role of negative urgency. Participants were 517 emerging adults from across the United States (ages 18–29; *M* = 22.60, SD = 3.81; 76% female). Participants reported on parental differential support, weight–shape concerns, eating behaviors, and negative urgency. Using moderated‐mediation analyses, we found that weight–shape concerns mediated associations between differential support and eating behaviors. Unequal maternal support and paternal favoritism showed distinct patterns, suggesting that differential parental support may carry different relational meanings. Associations were stronger among individuals with high negative urgency. Together, the findings suggest that unequal treatment and emotional reactivity may play an important role in understanding disordered eating within family contexts.

1

Disordered eating behaviors, including dietary restriction, weight and shape concerns, binge eating, and compensatory behaviors, are common among emerging adults and represent a growing public health concern worldwide (Pacanowski et al. 2024; Peschel et al. 2024). A substantial proportion of young adults report engaging in binge eating and compensatory behaviors (Fischer et al. 2023; Nagata et al. 2018; Peschel et al. 2024). Although disordered eating has historically been studied primarily among women, emerging adulthood represents a sensitive developmental window for men as well (Cain et al. 2012).

Such behaviors are not confined to clinically diagnosed cases; they are also prevalent in community populations and among individuals of varying body weights. Recent large‐sample work using brief screening tools indicates that a substantial portion of emerging adults report disordered eating behaviors, with prevalence estimates ranging from approximately 23% to nearly 49% in college students (Pacanowski et al. 2024). Accordingly, focusing on nonclinical samples is critical, given that many individuals with pronounced weight and shape concerns fall below diagnostic thresholds for an eating disorder (May et al. 2006).

Although previous research has linked family experiences and family‐level processes to disordered eating and weight‐shape concerns during adolescence and emerging adulthood (Eisenberg et al. 2012; Langdon‐Daly and Serpell 2017; Loth et al. 2014), the broader familial context in which disordered eating develops during this developmental period remains incompletely understood. In particular, less is known about the extent to which parental behaviors contribute to weight‐shape concerns and related eating pathology in young adulthood. At the same time, individual characteristics may shape vulnerability to disordered eating, suggesting that both contextual and dispositional factors warrant consideration (Fischer et al. 2023). The present study addresses this gap by examining associations between parental behaviors and disordered eating outcomes, with the goal of informing prevention efforts during this critical developmental period.

### Parental Differential Treatment, Weight‐Shape Concerns, and Disordered Eating Behaviors

Family systems theory (Cox and Paley 1997) and a growing body of research indicate that families play a crucial role in psychological well‐being across development (e.g., Hochgraf et al. 2025; Jensen and Thomsen 2024). Prior empirical evidence suggests that family features, such as parental support and control, family‐based physical activity support, and general family functioning, can increase vulnerability to, or buffer against, psychological and behavioral health difficulties (May et al. 2006; Wang et al. 2019).

In addition, a variety of parental behaviors, including criticism, weight‐related messages, and support, have been linked to weight–shape concerns and disordered eating behaviors (Berge et al. 2014; King et al. 2022; Langdon‐Daly and Serpell 2017). Although previous research has focused largely on parental behaviors directed toward individual children, the current study examines parental differential treatment, which captures how children evaluate parental behaviors relative to those directed toward a sibling. Parental differential treatment refers to the extent to which parents treat their children differently in areas such as affection, control, privileges, and support (McHale et al. 2000). Differential support may be particularly relevant to eating‐related concerns because it reflects the allocation of emotional and instrumental resources within the family and may influence perceptions of parental investment and acceptance.

Parental differential treatment may represent a unique family process that involves social comparison and perceptions of one's relative standing within the family. Unequal parental treatment may convey implicit messages about siblings' relative value and importance, potentially shaping self‐evaluative processes that have been implicated in the development of eating pathology (Langdon‐Daly and Serpell 2017; Loth et al. 2014). As a result, parental differential treatment may be an important family characteristic for understanding vulnerability to eating pathology. Despite these potential implications, parental differential treatment has received relatively little attention in the eating disorders literature.

According to equity theory (Adams 1963), individuals evaluate their inputs and outcomes relative to those of others. When applied to family relationships, equitable distribution of parental resources is typically perceived as ideal, whereas unequal allocation of parental resources may evoke distress for both favored and disfavored children (Polk 2022). Disadvantaged positions may foster feelings of unworthiness whereas advantaged positions may generate psychological strain due to heightened expectations and role pressures. Empirical research further indicates that unequal treatment, regardless of who receives more parental support, affection, or other resources, has been linked to poorer well‐being in emerging adulthood (Jensen and Thomsen 2024). Such dynamics may heighten sensitivity to evaluation and comparison within the family context, fostering self‐evaluative concerns and conditional self‐worth processes closely linked to weight and shape concerns (Murphy et al. 2016). Accordingly, the lowest levels of weight–shape concerns and disordered eating behaviors may be expected when parental support is perceived as relatively equal, whereas deviations from equal treatment in either direction may be associated with greater risk.

From a cognitive‐behavioral perspective (Williamson et al. 2004), feelings of unworthiness, guilt, or pressure to maintain perfection may foster the overvaluation of weight and shape, thereby increasing vulnerability to disordered eating attitudes. Consistent with this framework, individuals who perceive their parents as rejecting or controlling report elevated weight and eating concerns (Hochgraf et al. 2025; King et al. 2022) and greater engagement in disordered eating behaviors (Berge et al. 2014; King et al. 2022). Moreover, individuals formally diagnosed with eating disorders have reported experiences of parental over‐ or under‐involvement and favoritism within the family (Machado et al. 2020; Murphy et al. 2000; Wonderlich et al. 1994). Furthermore, Miles‐McLean and colleagues (2014) revealed that individuals with overprotective parents were vulnerable to developing negative eating attitudes.

To cope with heightened weight‐ and shape‐related concerns, individuals may employ various regulatory strategies. One such strategy involves shifting attention away from distressing self‐evaluations toward immediate sensory experiences (Heatherton and Baumeister 1991). In this context, binge eating may temporarily reduce aversive body‐related self‐awareness. Prior research supports associations between weight–shape concerns and binge eating (Trompeter et al. 2023). Compensatory behaviors often follow binge episodes (Williamson et al. 2004) and may function to restore perceived control over‐eating or alleviate anxiety and discomfort related to body image (Heatherton and Baumeister 1991).

Taken together, these perspectives suggest that parental differential treatment may contribute to heightened weight–shape concerns, which in turn increases vulnerability to binge eating and compensatory behaviors. Specifically, perceived differential treatment may shape internalized evaluations of self‐worth, which become expressed in the overvaluation of weight and shape. However, not all individuals exposed to differential treatment may respond in the same way, highlighting the importance of considering individual difference factors that may shape these associations (Gozu and Newman 2020; Jensen et al. 2020).

### The Moderating Role of Negative Urgency

Individuals exposed to perceived inequity or relational distress may differ in how they behaviorally respond to such experiences. One individual characteristic that has been consistently linked to binge eating and compensatory behaviors is negative urgency. Negative urgency refers to a tendency to act impulsively when experiencing intense negative emotions, particularly in emotionally charged situations (Cyders and Smith 2008). Among the broader dimensions of impulsivity, negative urgency has emerged as a particularly robust predictor of disordered eating behaviors (Fischer et al. 2023; Van Swearingen and Noel 2024).

Individuals high in negative urgency may be especially vulnerable during moments of distress. When emotions feel overwhelming, engaging in binge eating can provide short‐term relief, making it a compelling, though ultimately maladaptive, strategy for regulating affect (Emery et al. 2013; Ralph‐Nearman et al. 2020). This tendency toward rash action under emotional strain is also associated with greater engagement in compensatory behaviors (Davis et al. 2020; Van Swearingen and Noel 2024).

Importantly, negative urgency may not operate independently but instead intensifies the impact of emotionally painful experiences, such as body shame. Prior research indicates that impulsivity more broadly interacts with body‐related distress to predict binge‐type eating behaviors (Higgins et al. 2015), and that negative urgency strengthens the association between shame‐related experiences and maladaptive eating (Dalley et al. 2020). Extending this framework, negative urgency may amplify the likelihood that weight–shape concerns translate into maladaptive behavioral responses.

### The Present Study

In summary, prior research consistently demonstrates that weight–shape concerns are associated with binge eating and compensatory behaviors and that negative urgency may intensify these associations. Although general parental behaviors such as rejection, control, and overinvolvement have been linked to eating pathology, less is known about how parental differential treatment, particularly differential support between siblings, may contribute to the development of weight–shape concerns and related disordered eating behaviors. Understanding how parental differential support relates to weight–shape concerns and disordered eating behaviors may have implications for prevention and intervention efforts. Identifying family processes associated with eating‐related difficulties may help clinicians and families recognize relational dynamics that contribute to risk and inform strategies for fostering supportive family environments.

The current study examines associations among maternal and paternal differential support, weight–shape concerns, and binge eating and compensatory behaviors in emerging adulthood. Furthermore, we test whether negative urgency moderates the pathway linking weight–shape concerns to disordered eating behaviors (see Figure 1).

Hypothesis 1 Maternal and paternal differential support will be curvilinearly associated with eating behaviors (binge eating and compensatory behaviors) in emerging adulthood.

Hypothesis 2 Maternal and paternal differential support will be curvilinearly associated with weight–shape concerns.

Hypothesis 3 Weight–shape concerns will mediate the association between maternal and paternal differential support and eating behaviors (binge eating and compensatory behaviors).

Hypothesis 4 Negative urgency will moderate the second‐stage pathway between weight–shape concerns and eating behaviors, resulting in a conditional indirect association between parental differential support and eating behaviors (binge eating and compensatory behaviors) across levels of negative urgency.

**Figure 1:**
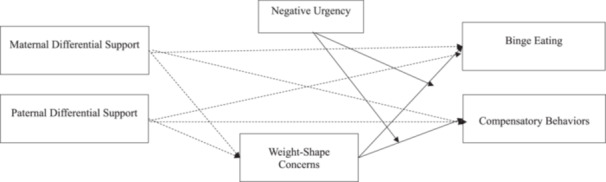
Parental differential support and binge eating and compensatory behaviors moderated‐mediation through weight‐shape concerns. *Note*: The dotted line indicates a *U*‐shaped relationship between variables.

## 2 Methods

### 2.1 Participants

The sample included 517 emerging adults residing in the United States, ranging in age from 18 to 29 years (*M* = 22.60, SD = 3.81). Most participants identified as female (76%), with 16% male, and the remainder identifying as nonbinary. The sample was racially and ethnically diverse, with 63% identifying as Caucasian, 13% Asian American, 8% Black/African American, 8% Hispanic American, and 7% reporting other identities. Participants reported varying birth order positions (44% oldest, 21% middle, and 35% youngest). Regarding family structure during most of their upbringing, 78% of participants reported living with two biological parents, 9% with a single mother, 7% with a biological mother and stepfather, and the remaining participants reported other family configurations.

Participants selected one target sibling defined as the sibling closest in age who did not have a serious ongoing childhood physical or mental health condition requiring treatment. Most target siblings were full biological siblings (89%), with smaller proportions of half‐, step‐, or adopted siblings. Slightly more than half of the dyads were mixed‐gender (55%), with the remainder same‐gender.

### 2.2 Procedure

Once the university's Institutional Review Board approved the study, participants were recruited to complete an online self‐report questionnaire. To be eligible, participants were required to be between 18 and 29 years of age and have at least one sibling. Participants were informed that the study examined family relationships, sibling experiences, and health‐related attitudes and behaviors. They were also informed that participation was voluntary, responses would remain confidential, and the survey would require approximately 20 min to complete.

Participants were recruited using two methods. First, faculty at a private urban university in the Northeastern United States informed students about the study. After providing informed consent, interested students completed the questionnaire either during a scheduled class session or independently outside of class. Second, additional participants were recruited through ResearchMatch (n.d.), a national health volunteer registry developed by multiple academic institutions and supported by the National Institutes of Health through the Clinical and Translational Science Award (CTSA) program.

## 3 Measures

### 3.1 Maternal and Paternal Differential Treatment

We measured perceived maternal and paternal differential treatment using an adapted version of the Intergenerational Support Index (Fingerman et al. 2009). This 10‐item scale assesses emotional, practical, communication, advice‐giving, and financial support (e.g., “My mother/father helps _____ think about a problem.”). Participants rated each item on a 5‐point scale (1 = *my sibling a lot more*, 3 = *both of us equally*, 5 = *me a lot more*). The average across items indexed overall support scores for mothers and fathers. All Cronbach's alpha coefficients indicated satisfactory internal consistency in the current sample (Cronbach's *α* = 0.82 for maternal differential support; Cronbach's *α* = 0.83 for paternal differential support, see Table 1). The average score (1–5) reflects both the direction and magnitude of differential support, such that scores above the midpoint (3) indicate that the participant received more parental support, whereas scores below the midpoint indicate that the sibling received more support.

**Table 1:** Correlations and descriptive statistics.

Variable	*M*	SD	1	2	3	4	5	6
1. Maternal Differential Support	2.94	0.57	(0.82)					
2. Paternal Differential Support	3.00	0.54	0.39***	(0.83)				
3. Weight‐Shape Concerns	3.04	1.75	0.02	0.06	(0.85)			
4. Negative Urgency	2.30	.69	−0.04	0.16**	0.23***	(0.79)		
5. Binge Eating	6.39	11.04	0.01	0.06	0.45***	0.20***	NA	
6. Compensatory Behaviors	2.33	5.95	0.01	0.11*	0.40***	0.16***	0.32***	NA

*Note: N* ranges from 487 to 517 due to missing data. Cronbach's *α*s are in the parentheses. For binge eating and compensatory behavior, internal consistency reliability indices (e.g., Cronbach's *α*) are not applicable. Reliability estimates were not computed for binge eating and compensatory behavior variables because they represent behavioral frequency counts rather than multi‐item scales.

* *p* < 0.05

** *p* < 0.01

*** *p* < 0.001.

### 3.2 Weight and Shape Concerns and Eating Behaviors

We assessed participants' weight‐shape concerns using five items from the Eating Disorder Examination Questionnaire (EDE‐Q; Fairburn and Beglin 1994). We selected these items a priori to capture the core cognitive–evaluative features of weight‐shape concerns most relevant to our hypotheses while minimizing participant burden in a multi‐construct survey. The items assessed fear of weight gain, negative evaluations of body shape and weight, and preoccupation with weight‐ and shape‐related thoughts (e.g., “Have you felt fat?”). Participants reported how often they experienced each symptom over the past 28 days using a 7‐point scale ranging from 0 days (none) to every day (28 days). Although the items come from two EDE‐Q subscales (Weight Concern and Shape Concern), we combined them into a single composite reflecting overall weight‐shape concerns, consistent with prior research treating these domains as a unified construct (e.g., Bardone‐Cone et al. 2008). Consistent with previous applications of the measure, item responses were averaged to create a composite score, with higher scores indicating greater weight‐shape concerns (Cronbach's *α* = 0.85; see Table 1).

The frequency of objective eating behaviors was assessed using EDE‐Q items measuring binge eating (three items) and compensatory behaviors (three items). Participants reported the number of days in the past 28 days on which they experienced binge eating episodes, defined as episodes involving an unusually large amount of food accompanied by a perceived loss of control, as well as the number of days on which they engaged in compensatory behaviors (self‐induced vomiting, laxative use, compulsive exercise) for weight or shape control. Responses were summed across the 28‐day reporting period to create total binge eating and compensatory behavior frequency scores, with higher scores indicating more frequent engagement in these behaviors. Reliability estimates were not computed for binge eating and compensatory behaviors because they represent behavioral frequency counts derived from single EDE‐Q items rather than multi‐item scales.

### 3.3 Negative Urgency

We assessed negative urgency using the Negative Urgency subscale from the short form of the Urgency, Premeditation (lack of), Perseverance (lack of), Sensation Seeking, and Positive Urgency Impulsive Behavior Scale (UPPS‐P; Cyders et al. 2014). Items assess the tendency to act rashly in response to intense negative emotions (e.g., “When I feel rejected, I will often say things that I later regret”). Participants rated each item on a 4‐point Likert scale ranging from 1 (strongly agree) to 4 (strongly disagree). We averaged item responses to create a composite score, with higher scores indicating greater negative urgency (*α* = 0.79).

### 3.4 Covariates

Previous research shows that gender, age, family size, and maternal and paternal educational attainment are associated with binge eating and compensatory behaviors as well as weight‐shape concerns (Cain et al. 2012; Kim 2016; Ragelienė and Grønhøj 2020; Vandereycken and Van Vreckem 1992). Accordingly, we included gender (man vs. woman; nonbinary person vs. woman) as dummy‐coded control variables, and treated family size, age, maternal and paternal educational attainment as continuous covariates.

## 4 Data Analysis Strategy

### 4.1 Model Fit

Fit indices included the Chi‐square (*χ* ^2^), Comparative Fit Index (CFI), Tucker‐Lewis Index (TLI), Root Mean Square Error of Approximation (RMSEA), and Standardized Root Mean Square Residual (SRMR). CFI and TLI values above 0.95 and SRMR and RMSEA values below 0.08 indicate acceptable to good model fit (Kline 2016).

### 4.2 Mediation Analysis

Because differential support may relate to outcomes in nonlinear ways, we included both linear and quadratic terms for maternal and paternal differential support (Dawson 2014). This approach allowed us to test whether relatively lower or higher levels of support (compared to a sibling) were associated with weight‐shape concerns. Because nonlinear models allow indirect effects to vary across values of the predictor, we examined instantaneous indirect effects. Unlike traditional mediation analyses, which estimate a single indirect effect, instantaneous indirect effects allow the magnitude and direction of the indirect effect to vary across values of the predictor when quadratic terms are included in the model (Hayes and Preacher 2010).

We tested the hypothesized mediation model using path analysis within a structural equation modeling framework in IBM SPSS AMOS 31.0 (Arbuckle 2007). Parameters were estimated using maximum likelihood estimation. Prior to analysis, missing data were addressed using regression imputation in AMOS, and the imputed data set was subsequently used in all analyses. Maternal and paternal differential support (linear and quadratic terms) were simultaneously entered as predictors of weight‐shape concerns and eating behaviors (binge eating and compensatory behaviors).

We evaluated instantaneous indirect effects using bias‐corrected bootstrapping with 5000 resamples and generated unstandardized indirect effect estimates with 95% confidence intervals (Hayes and Preacher 2010). We considered mediation supported when confidence intervals did not include zero (Shrout and Bolger 2002).

To clarify nonlinear effects, we estimated instantaneous indirect effects at −1, 0, and 1 levels of parental differential support. These values represented relatively greater support toward the sibling (−1), equal support (0), and greater support toward the participant (1). This approach allowed us to evaluate how associations varied across conceptually meaningful levels of perceived differential support.

### 4.3 Moderated‐Mediation Analysis

We tested a second‐stage moderated‐mediation model in which negative urgency moderated the association between weight–shape concerns and eating behaviors. Before creating interaction terms, we mean‐centered all continuous variables. We then included an interaction term between weight–shape concerns and negative urgency in predicting binge eating and compensatory behaviors.

We estimated conditional instantaneous indirect effects of parental differential support on eating behaviors through weight–shape concerns at low and high levels of negative urgency using bias‐corrected bootstrapping with 5000 resamples. We concluded that moderated mediation was present when the conditional indirect effects varied across levels of negative urgency and the corresponding confidence intervals excluded zero.

## 5 Results

### 5.1 Model Fit

Among the covariates examined, gender, maternal and paternal educational attainment were significantly correlated with the mediator and outcome variables and were therefore retained in the analyses. We then conducted path analyses in AMOS to examine whether weight‐shape concerns mediated the associations between maternal and paternal differential support and binge eating and compensatory behaviors, while negative urgency moderated the links between weight‐shape concerns and binge eating and compensatory behaviors. The model demonstrated acceptable fit to the data, ML *χ* ^2^ (12) = 40.03, *p* < 0.001, CFI = 0.97, TLI = 0.79, RMSEA = 0.067 95% CI [0.045, 0.091], SRMR = 0.03, although the TLI fell below typical benchmarks. Results below are reported from the final analytic model, which retained all theoretically relevant paths; nonsignificant paths are noted where appropriate.

Hypothesis 1 Associations between maternal and paternal differential support and eating behaviors.

As shown in Table 1, maternal differential support was not associated with binge eating or compensatory behaviors at the bivariate level, whereas paternal differential support was positively correlated with compensatory behaviors only (*r* = 0.11, *p* < 0.05). When all variables, including the mediator and linear and quadratic terms for parental differential support, were entered into the model (see Table 2), the linear term for maternal differential support remained nonsignificant, whereas the quadratic term emerged as a significant predictor of compensatory behavior, such that compensatory behaviors were lowest when maternal support was perceived as equal and higher when maternal support was directed toward either the participant or the sibling (*B* = 1.08, SE = 0.45, *β* = 0.11, *p* = 0.016), indicating a U‐shaped relationship. In contrast, neither the linear nor the quadratic terms for paternal differential support significantly predicted binge eating or compensatory behaviors.

**Table 2:** Summary of moderated mediation model of maternal and paternal differential support (MDS, PDS) on binge eating and compensatory behaviors through weight‐shape concerns.

		*B*	SE	*β*	*p*
Predicting Weight‐Shape Concerns	MDS → Weight‐Shape Concerns	0.02	0.16	0.01	0.904
	${\text{MDS}}^{2}$ → Weight‐Shape Concerns	0.31	0.14	0.12	0.023
	PDS → Weight‐Shape Concerns	0.52	0.16	0.15	0.001
	${\text{PDS}}^{2}$ → Weight‐Shape Concerns	0.03	0.14	0.01	0.831
Predicting Binge Eating	MDS → Binge Eating	0.78	0.94	0.04	0.409
	${\text{MDS}}^{2}$ → Binge Eating	0.95	0.82	0.05	0.246
	PDS → Binge Eating	−1.16	0.97	−0.05	0.229
	${\text{PDS}}^{2}$ → Binge eating	−0.45	0.84	−0.02	0.592
	Weight‐Shape Concerns → Binge Eating	2.79	0.26	0.43	< 0.001
	Negative Urgency → Binge Eating	1.57	0.68	0.09	0.021
	Weight‐Shape Concerns × Negative Urgency → Binge Eating	0.19	0.37	0.02	0.597
Predicting Compensatory Behaviors	MDS → Compensatory Behaviors	0.20	0.52	0.02	0.708
	${\text{MDS}}^{2}$ → Compensatory Behaviors	1.08	0.45	0.11	0.016
	PDS → Compensatory Behaviors	0.38	0.53	0.03	0.320
	${\text{PDS}}^{2}$ → Compensatory Behaviors	−0.59	0.46	−0.07	0.198
	Weight‐Shape Concerns → Compensatory Behaviors	1.26	0.14	0.36	< 0.001
	Negative Urgency → Compensatory Behaviors	0.37	0.37	0.04	0.320
	Weight‐Shape Concerns × Negative Urgency → Compensatory Behaviors	0.48	0.20	0.10	0.018

*Note:* *p < 0.05, **p < 0.01, ***p < 0.001.

Hypothesis 2 Associations among maternal and paternal differential support and weight–shape concerns.

Maternal differential support exhibited a significant curvilinear association with weight–shape concerns. Specifically, the quadratic term for maternal differential support significantly predicted weight–shape concerns, such that concerns were lowest when maternal support was perceived as equal and higher when maternal support was directed toward either the participant or the sibling (*B* = 0.31, SE = 0.14, *β* = 0.12, *p* = 0.023), indicating a U‐shaped relationship. This pattern suggests that weight‐shape concerns were lowest when maternal support was relatively equal and increased as maternal support became more differential, regardless of whether the participant or the sibling received greater support.

In contrast, paternal differential support showed a significant linear association with weight‐shape concerns (*B* = 0.52, SE = 0.16, *β* = 0.15, *p* < 0.001). This finding indicates that greater paternal support directed toward the participant (relative to the sibling) was associated with higher levels of weight‐shape concerns.

Hypothesis 3 Weight–shape concerns as a mediator of the association between parental differential support and eating behaviors.

We examined whether weight–shape concerns mediated the associations between parental differential support and eating behavioral symptoms. Weight–shape concerns significantly predicted both binge eating (*B* = 2.79, SE = 0.26, *β* = 0.43, *p* < 0.001) and compensatory behaviors (*B* = 1.26, SE = 0.14, *β* = 0.36, *p* < 0.001; see Table 2). Instantaneous indirect effects were estimated using bias‐corrected bootstrapping with 5000 resamples and were probed at levels of maternal differential support below the mean, at the mean, and above the mean, corresponding to situations in which the sibling received relatively more support, parental support was equal, and the participant received relatively more support, respectively. Because the instantaneous indirect effect was moderated by negative urgency for compensatory behaviors but not for binge eating, results from the same analytic model are presented in separate tables for clarity.

For binge eating, as shown in Table 3A, weight–shape concerns mediated the association between maternal differential support and binge eating. When the sibling received relatively greater maternal support, movement toward more equal treatment was associated with lower levels of binge eating through lower weight–shape concerns ($\emptyset_{x=0}$ = −1.71, 95% CI [−3.44, −0.35]). At the mean level of maternal differential support (i.e., equal support), the instantaneous indirect effect was not significant. In contrast, further increases in maternal support directed toward the participant were associated with higher levels of binge eating through elevated weight–shape concerns ($\emptyset_{x=1}$ = 3.47, 95% CI [0.25, 7.48]). Because the 95% bias‐corrected confidence intervals did not include zero, these instantaneous indirect effects were statistically significant (Shrout and Bolger 2002). This pattern indicates that equal maternal support was associated with lower weight–shape concerns and, consequently, lower levels of binge eating, whereas maternal support favoring either the participant or the sibling was associated with elevated weight–shape concerns and higher levels of binge eating.

**Table 3A:** Direct, linear, and instantaneous indirect effects of maternal and paternal differential support on binge eating from mediation analysis.

Predictor	Level of PDT	Direct	Indirect	Lower	Upper
MDS	Below the mean	−1.12	−1.71*	−3.44	−0.35
	The mean	0.78	0.02	−0.31	0.40
	Above the mean	2.68	3.47*	0.25	7.48
PDS	Linear (overall)	−0.45	1.45**	0.57	2.57

*Note:* Unstandardized coefficients are based on 5000 bootstrap estimates. The direct effect of MDS is defined as $c_{1}+2c_{2}X$. The instantaneous indirect effect of MDS is defined as $\emptyset =\left(a_{1}+2a_{2}X\right)b$. X = maternal differential support (MDS) and M = Weight‐Shape Concerns; paternal differential support = PDS.

* *p* < 0.05

** *p* < 0.01

*** *p* < 0.001.

Table 3A also shows that the linear indirect effect of paternal differential support was significant (*B* = 1.45, 95% CI [0.57, 2.57]). This indicates that greater paternal support directed toward the participant was associated with higher levels of binge eating through elevated weight–shape concerns.

For compensatory behaviors, as shown in Table 3B, weight–shape concerns mediated the association between maternal differential support and compensatory behaviors. When the sibling received relatively greater maternal support, movement toward more equal treatment was associated with lower levels of compensatory behaviors through weight–shape concerns ($\emptyset_{x=0}$ = −0.76, 95% CI [−1.51, −0.18]). At the mean level of maternal differential support and at high levels of maternal support directed toward the participant, the instantaneous indirect effects were not significant. This pattern suggests that when maternal support favored the sibling, higher levels of compensatory behaviors were observed through elevated weight–shape concerns. As maternal support became more equal, compensatory behaviors decreased. However, once maternal support reached relatively equal levels, further shifts toward participant‐favored support were not associated with meaningful changes in compensatory behaviors through weight–shape concerns.

**Table 3B:** Direct, linear, and conditional instantaneous indirect effects of maternal and paternal differential support on compensatory behaviors from moderated‐mediation analysis.

		Mediation				Moderated‐mediation							
						Low negative urgency indirect				High negative urgency indirect			
		Direct	Indirect	95% CI LL	95% CI UL	Direct	Indirect	95% CI LL	95% CI UL	Direct	Indirect	95% CI LL	95% CI UL
MDS	Level of PDT												
	Below the mean	−1.96	−0.76*	−1.51	−0.18	−1.96	−0.47*	−1.23	−0.03	−1.96	−1.04*	−2.29	−0.28
	The mean	0.20	0.02	−0.39	0.54	0.20	0.02	−0.26	0.42	0.20	0.03	−0.55	0.73
	Above the mean	2.36	0.81	−0.02	2.19	4.52	0.99	−0.00	3.31	2.36	2.19*	0.12	5.90
PDS	Linear (overall)	0.59	0.66**	0.26	1.20	0.59	0.41*	0.05	0.98	0.59	0.91**	0.38	1.90

*Note:* Unstandardized coefficients are based on 5000 bootstrap estimates. The direct effect of MDS is defined as $c_{1}+2c_{2}X$. The instantaneous indirect effect of MDS is defined as $\emptyset =\left(a_{1}+2a_{2}X\right)b$, while the conditional instantaneous indirect effect of MDS is defined as $\emptyset =\left(a_{1}+2a_{2}X\right)(b_{1}+b_{2\;}W)$. *X* = MDS, *M* = weight‐shape concerns, *W* = negative urgency; paternal differential support = PDS.

* *p* < 0.05

** *p* < 0.01

** *p* < 0.001.

Table 3B further shows that the linear indirect effect of paternal differential support was significant (*B* = 0.66, 95% CI [0.26, 1.20]). This indicates that greater paternal support directed toward the participant was associated with higher levels of compensatory behavior through elevated weight–shape concerns.

Hypothesis 4 Moderation of the indirect association by negative urgency.

Negative urgency moderated the second‐stage pathway between weight–shape concerns and compensatory behaviors, such that the indirect effect of maternal differential support on compensatory behaviors through weight–shape concerns depended on participants' levels of negative urgency. For maternal differential support, we estimated conditional indirect effects at low, mean, and high levels of differential support, separately at low and high levels of negative urgency.

When the sibling received relatively greater maternal support, movement toward more equal treatment was associated with lower weight–shape concerns, which in turn were associated with lower levels of compensatory behavior at both low negative urgency ($\emptyset_{x=0}$ = −0.47, 95% CI [−1.23, −0.03]) and high level of negative urgency ($\emptyset_{x=1}$ = −1.04, 95% CI [−2.29, −0.28]). However, as shown in Table 3B, this decline was stronger among participants with high negative urgency than those with low negative urgency.

At the mean level of maternal differential support (i.e., relatively equal support), the conditional instantaneous indirect effects through weight–shape concerns were not significant for participants with either low or high negative urgency. In contrast, further increases in maternal support directed toward the participant were associated with higher levels of compensatory behaviors through elevated weight–shape concerns, but only among participants with high negative urgency ($\emptyset_{x=1}$ = 2.19, 95% CI [.12, 5.90]).

As shown in Table 3B, the conditional linear indirect effect of paternal differential support was also significant. Greater paternal support directed toward the participant was associated with higher levels of compensatory behaviors through elevated weight–shape concerns, at low levels of negative urgency (*B* = 0.41, 95% CI [0.05, 0.98]) and high negative urgency (*B* = 0.91, 95% CI [0.38, 1.90]). Participants with high negative urgency exhibited a stronger increase in compensatory behaviors through elevated weight‐shape concerns compared to those with low negative urgency.

## 6 Discussion

The present findings suggest that parental differential support may represent an important family context associated with disordered eating in emerging adulthood. Weight–shape concerns emerged as a key mechanism linking parental differential support to binge eating and compensatory behaviors, and these associations varied by parent and by individuals' levels of negative urgency. This pattern highlights the complex ways family experiences and individual characteristics may jointly contribute to eating‐related difficulties.

### 6.1 Weight–Shape Concerns as a Mechanism Linking Differential Support and Disordered Eating

Weight–shape concerns emerged as a key mechanism linking parental differential support to disordered eating behaviors in the current study. Consistent with cognitive‐behavioral models of disordered eating (Williamson et al. 2004), weight–shape concerns helped explain links between parental differential support and both binge eating and compensatory behaviors. When individuals perceived deviations from equal maternal support, as well as greater paternal support directed toward themselves relative to their sibling, they reported higher binge eating indirectly through increased weight–shape concerns. In contrast, perceiving less maternal support relative to one's sibling was indirectly associated with compensatory behaviors through heightened weight–shape concerns. Greater paternal support directed toward the participant was also indirectly associated with compensatory behaviors through the same pathway. The pattern of indirect effects mirrored these parent gender differences.

In line with escape theory (Heatherton and Baumeister 1991), individuals experiencing heightened distress may turn to binge eating to momentarily step away from negative emotions related to weight–shape concerns. At the same time, heightened weight–shape concerns may increase the likelihood of engaging in compensatory behaviors, which may function as attempts to “undo” or regulate perceived shortcomings. Together, these findings suggest that everyday relational experiences within the family may shape how individuals evaluate themselves, which in turn can increase vulnerability to different forms of disordered eating. These results are consistent with prior research linking family dynamics and body‐related concerns to disordered eating outcomes (Berge et al. 2014; King et al. 2022).

### 6.2 Maternal and Paternal Differential Support

Maternal differential support showed a curvilinear association with compensatory behaviors. Emerging adults who perceived receiving either more or less maternal support than their sibling reported higher levels of compensatory behaviors, whereas those who perceived maternal support as relatively equal reported the lowest levels. This pattern suggests that unequal maternal support, regardless of direction, may carry psychological costs. In line with equity theory (Polk 2022), feeling disfavored may evoke rejection or unworthiness, whereas feeling favored may generate guilt or pressure to live up to expectations. In either case, relational inequity may contribute to vulnerability in eating‐related behaviors.

With respect to weight–shape concerns, maternal and paternal support demonstrated distinct patterns. Maternal differential support followed a curvilinear association, whereas paternal differential support was linearly associated with these concerns. These differences suggest that maternal and paternal support may carry different relational meanings during emerging adulthood. Maternal support may be more closely tied to perceived relational status and one's sense of worth within the family; thus, deviations from equity may increase self‐evaluative concerns (e.g., Shebloski et al. 2005). Because maternal relationships often serve as important sources of emotional support and validation, differences in maternal support may be particularly salient indicators of acceptance and belonging within the family system (Laursen and Collins 2009). In contrast, greater paternal support relative to a sibling may be interpreted as heightened expectations or performance pressure. Under these circumstances, paternal favoritism may be experienced not only as support but also as an expectation to justify or live up to preferential treatment. Although speculative, these differing interpretations may help explain why maternal and paternal differential support demonstrated distinct associations with weight–shape concerns. Together, these findings extend prior work linking parental involvement and favoritism to disordered eating by highlighting the importance of relative support within the sibling context.

### 6.3 Differential Pathways to Binge Eating and Compensatory Behaviors

Although maternal differential support showed a curvilinear direct association with compensatory behaviors, the mediated pathways through weight–shape concerns revealed a more asymmetric pattern. Whereas unequal maternal support in either direction was directly associated with higher compensatory behaviors, the indirect effect through weight–shape concerns emerged only when individuals perceived receiving less maternal support than their sibling. In contrast, general deviations from equal maternal support, regardless of direction, were indirectly associated with binge eating via elevated weight–shape concerns. These findings suggest that while unequal maternal support broadly relates to compensatory behaviors at the direct level, self‐evaluative concerns about weight and shape may be particularly activated under conditions of maternal disfavoritism. Perceiving oneself as receiving less support than a sibling may heighten feelings of inadequacy, rejection, or diminished relational value, which may become reflected in negative evaluations of one's body and appearance. Thus, perceiving oneself as disfavored may heighten weight–shape concerns, thereby increasing vulnerability to compensatory behaviors.

### 6.4 The Moderating Role of Negative Urgency

Importantly, the asymmetric indirect pattern described above was conditional on levels of negative urgency. Among individuals with low negative urgency, the indirect association between maternal differential support and compensatory behaviors remained asymmetric, emerging primarily under conditions of perceived maternal disfavoritism. However, among individuals with high negative urgency, the indirect effect became more symmetric, such that deviations from equal maternal support in either direction were associated with higher compensatory behaviors via weight–shape concerns.

For paternal differential support, a somewhat different pattern emerged. Although greater paternal support directed toward the participant (i.e., favoritism) was indirectly associated with compensatory behaviors through weight–shape concerns across levels of negative urgency, this association was amplified among individuals high in negative urgency. This suggests that heightened emotional reactivity may amplify sensitivity not only to being disfavored but also to being favored. When emotions feel intense and overwhelming, those high in negative urgency may be especially likely to respond quickly to manage distress or regain a sense of control (Dalley et al. 2020). This moderation did not emerge for binge eating, suggesting that these behaviors may arise from partially distinct emotional processes. Binge eating may function more to escape distress, whereas compensatory behaviors may reflect more immediate, urgency‐driven attempts to regulate it.

### 6.5 Limitations and Suggestions for Future Research

The current study offers insight into how parental differential treatment may relate to disordered eating through weight–shape concerns, particularly in the context of negative urgency. At the same time, several limitations should be considered when interpreting the findings.

First, although we assessed participants' concerns about weight and shape, we did not collect data on body mass index (BMI). Including BMI in future research would help clarify whether these associations hold beyond objective weight status. In addition, other family‐related influences, such as parental comments about weight or shared family meal practices (Dahill et al. 2022), were not measured in the current study. These contextual factors may also play an important role in shaping eating‐related outcomes and would be valuable to examine in future work.

Second, the cross‐sectional design limits our ability to draw conclusions about directionality. Longitudinal research following families over time would provide a clearer picture of how parental differential treatment and weight–shape concerns may unfold and interact across development. Third, the generalizability of the findings is limited. The sample was predominantly White (63%) and female (76%), which may have influenced both the observed findings and their applicability to more diverse populations. Given that women generally report higher levels of weight‐shape concerns and disordered eating behaviors than men, the present findings may be more representative of women's experiences (Berge et al. 2014). In addition, associations between parental differential support and eating‐related outcomes may vary as a function of participant gender and parent‐child gender dynamics, which were not directly examined in the present study. Replicating this model with more racially and ethnically diverse samples, as well as greater representation of males and gender‐diverse individuals, would strengthen confidence in the findings and enhance their generalizability.

Furthermore, family structure may influence the meaning and impact of parental differential support. However, the present sample was predominantly composed of participants who reported living with two biological parents during childhood. Future research should examine whether associations between differential support, weight‐shape concerns, and disordered eating differ across family structures, including single‐parent, blended, and adoptive families. Future research should also examine sibling gender composition. Because same‐gender siblings may be more likely to compare themselves to one another, parental differential support may be more salient and influential in same‐gender sibling dyads than in mixed‐gender sibling dyads.

Finally, weight–shape concerns were assessed using five items drawn from the original scale. Although internal consistency in the present sample was strong (*α* = 0.85), the use of a shortened measure may not fully capture the multidimensional nature of the construct. Future studies should consider using the full scale to provide a more comprehensive assessment.

### 6.6 Implications

Our findings suggest that how parental support is distributed and perceived within the sibling context may be linked to emerging adults' well‐being. Unequal maternal support and greater paternal support were associated with higher weight–shape concerns and disordered eating. Such findings underscore that both feeling disfavored and favored can carry emotional costs. These dynamics may be especially salient for emerging adults with high negative urgency, who may be more prone to coping with distress through dysregulated eating. Parents may benefit from reflecting on how their support is experienced relative to siblings and from fostering open conversations about fairness, expectations, and comparison within the family.

These findings also underscore the value of considering both family dynamics and individual vulnerabilities in prevention and intervention efforts. Mental health professionals working with emerging adults may find it helpful to explore perceptions of parental differential treatment alongside traits such as negative urgency when addressing body‐related distress and eating behaviors. Targeting both relational patterns and emotion‐driven impulsivity may provide a more comprehensive approach to supporting healthier self‐evaluations and eating behaviors.

## 7 Conclusion

In conclusion, these findings suggest that how parents differ in the support they provide to their children matters, not only how much support is given, but who receives more or less of it. Unequal maternal support and paternal favoritism carried distinct relational meanings, shaping weight–shape concerns and, in turn, different forms of disordered eating. Together, the results highlight that subtle differences in perceived support within the sibling context may relate to how young adults evaluate themselves, regulate distress, and engage in disordered eating.

## Funding

The authors have nothing to report.

## Ethics Statement

All procedures performed in studies involving human participants were conducted in accordance with the ethical standards of the institutional and/or national research committee and with the 1964 Helsinki Declaration and its later amendments or comparable ethical standards. Necessary approval was obtained from the Institutional Review Board of Thomas Jefferson University (iRIS ID‐2025‐0087).

## Consent

Informed consent was obtained from all individual participants included in the study.

## Data Availability

The data that support the findings of this study are available from the corresponding author upon reasonable request.
